# Effect of monovalent and bivalent live attenuated vaccines against QX-like IBV infection in young chickens

**DOI:** 10.1016/j.psj.2023.102501

**Published:** 2023-01-14

**Authors:** Chen-Yu Yang, Peng Peng, Xing Liu, Yongchang Cao, Yun Zhang

**Affiliations:** ⁎ The Brain Cognition and Brain Disease Institute (BCBDI), Shenzhen Institute of Advanced Technology, Chinese Academy of Sciences, Shenzhen 518055, China; † State Key Laboratory of Biocontrol, School of Life Sciences, Sun Yat-sen University, Guangzhou 510006, China

**Keywords:** IBV, QX-like (GI-19), young chicken, monovalent vaccine, bivalent vaccine

## Abstract

Since 1999, QX-like (GI-19) avian infectious bronchitis viruses have been the predominant strains in China till now. Vaccination is the most effective way to control the disease, while live attenuated vaccine is widely used. In the current research, we evaluated the effect of several monovalent and bivalent live IBV vaccines in young chickens against the QX-like (GI-19) IBV infection. The results showed that monovalent 4/91 and bivalent Ma5+LDT3 vaccines could provide efficient protection in day-old chickens that reduced morbidity and mortality, ameliorated histopathology lesions, and reduced viral loads were observed. These data suggest that vaccination through nasal route with monovalent 4/91 or bivalent Ma5+LDT3 in day-old chickens could serve a safe and effective vaccination strategy for controlling QX-like (GI-19) infectious bronchitis virus.

## INTRODUCTION

Avian infectious bronchitis (**IB**) is endemic in poultry industry worldwide. The causing pathogen avian infectious bronchitis virus (**IBV)** is a γ-coronavirus, infecting upper respiratory tract, reproductive system, and kidney of chicken (Cavanagh, 2007). Chickens of all ages are susceptible to IBV, and young chickens presented severer clinical signs compared with older ones (Animas et al., 1994).

The viral genome of IBV is a linear, single-stranded, positive-sense RNA with a length of approximately 27 kilobases, encoding four structural proteins (spike, envelope, matrix, and nucleocapsid) (Jordan, 2017). The spike (**S**) protein is further post-translationally cleaved into the amino-terminal S1 and the carboxyl-terminal S2 subunits (Cavanagh et al., 1986). According to the complete nucleotide sequences of the S1 gene, 6 genotypes comprising 32 distinct lineages (GI-1–GI-32) are defined (Valastro et al., 2016). In China, the QX-like (GI-19) IBV is one of the predominant IBV genotypes since 1999 (Zhao et al., 2017; Lian et al., 2021).

Prevention of any infection requires a high level of biosecurity, including cleaning and disinfection of the breeding environment, which is costly. Since IBV is highly infectious and prevalent, vaccination is generally considered to be the most effective and more economical approach for disease control, and live-attenuated vaccines are extensively used in the field. However, due to lack of cross-protection in most IBV commercial vaccines, vaccination with a single vaccine usually provide less protection against IBV strains with different serotypes (Gao et al., 2016). Though recombination of multiple vaccines covering different serotypes was reported to show broad protective spectrum (Zhao et al., 2015; Abdel-Sabour et al., 2021), the risk of recombination within live virus strains still exists. In order to identify better vaccination for day-old chickens, this study applied several heterologous monovalent and bivalent live attenuated vaccines and evaluated the protection conferred by these vaccines against the predominant QX-like (GI-19) IBV. The results suggest that vaccination through nasal route with monovalent 4/91 or bivalent Ma5 + LDT3 in day-old chickens could serve an effective vaccination strategy for controlling QX-like (GI-19) IBV infection.

## MATERIALS AND METHODS

### Vaccines and Virus Strains

Nobilis IB 4/91 (MSD Animal Health, Netherlands, Batch No.: A241A1J01), H120 (Sinder-Vet Techonology, China), LDT3 (Weike Biotechnology Development Co., Ltd., China) and Nobilis IB Ma5 (MSD Animal Health, China) were used for efficacy test and a field-isolated strain HSJ-2016 (Zhang et al., 2018) was used for challenge experiment.

### Efficacy Test

A total of 210 one-day-old SPF chickens were divided into 7 groups (30 birds/group) (Table 1). According to the instruction of the manufacturers, in the single-vaccinated groups (4/91, H120, LDT3), each bird was intranasally inoculated with 1 dose of Nobilis IB 4/91, Nobilis IB Ma5, LDT3, or H120, respectively. In the recombined-vaccinated groups (4/91 + Ma5, Ma5 + LDT3), each bird was intranasally inoculated with 1 dose of each vaccine, respectively.

**Table 1:** Efficacy test of monovalent and bivalent live vaccines against QX-like IBV.

Group	Vaccine	Number	Vaccination route and dosage	Challenge strain and dosage	Morbidity	Mortality	Protection
1	Nobilis IB 4/91	20 + 10	Eye drop 0.2 mL/bird	HSJ-2016 10^5.0^ EID_50_/bird	2/10	0/10	8/10
2	H120	20 + 10			4/10	0/10	6/10
3	LDT3	20 + 10			4/10	0/10	6/10
4	Nobilis IB 4/91 + Ma5	20 + 10			3/10	0/10	7/10
5	Ma5 + LDT3	20 + 10			2/10	0/10	8/10
6	Positive Control	20 + 10	-		9/9	1/10	0/10
7	Negative Control	20 + 10		-	0/10	0/10	-

All vaccinated birds were vaccinated at day one and kept in isolators with positive pressure in air-conditioned rooms. At 21 d of age, birds in 3 single-vaccinated groups, two combined-vaccinated groups and one positive control group (**PC**) were intranasally challenged with field strain HSJ-2016 (10^5.0^ EID_50_/bird) (Table 1). The negative control (**NC**) group was kept as negative control. The details of groups are shown in Table 1. Before challenge, sera of all birds were collected and antibodies were tested using commercial enzyme-linked immunosorbent assay (**ELISA**) (IDEXX, Westbrook, Maine) according to manufacturer's instruction. At 3- and 7-days post challenge (dpc), tracheas and kidneys of five chickens in each group were collected and processed for histopathology test and viral loads test. At 21 dpc, all birds were sacrificed for postmortem examination. During the experiment, morbidity and mortality of the chickens were evaluated.

### Histopathology

The trachea and kidney samples collected in the efficacy test were fixed in 10% formalin, routinely processed, and embedded in paraffin wax. Five micrometer thin sections were cut and stained with hematoxylin and eosin. The slides were examined with light microscopy for lesions.

### Real Time RT-PCR

To examine viral replication ability in the collected tracheas and kidneys, real time RT-PCR was performed as described before (Zhang et al., 2018). Briefly, cDNA was obtained by reverse transcription using a PrimerScript RT Master Mix Perfect Real Time kit (TaKaRa, Otsu, Shiga, Japan) according to the manufacturer's instruction and used later. Primers were designated using Primer Express 3.0 (Thermo Fisher Scientific, Waltham, MA) based on the conserved region of 1a gene to detect a 127-bp fragment (IBV-F: GCTTTTGAGCCTAGCGTT; IBV-R: GCCATGTTGTCACTGTCTATT) (Mo et al., 2020). The 20 μL PCR mixture was composed of 10 μL SYBR Premix EX TaqTM II (Tli RNaseH Plus) Kit (TaKaRa Bio, Mountain View, CA), 0.5 μmol of each primer, 0.4 μL ROX II, 100 ng cDNA template and 8 μL double-distilled water. Real-time PCR was performed on an Applied Biosystems 7500 Fast Real-Time PCR System (Thermo Fisher Scientific, Waltham, MA). Statistical data was converted to a linear form by the 2−CT calculation and the relative RNA copy numbers was analyzed by GraphPad Prism (GraphPad Software Inc., San Diego, CA). The CT values were obtained from each reaction containing the standard RNA with copies from 10^1^ to 10^7^. Actin was used as house-keeping gene (Villanueva et al., 2011). Viral loads in the collected samples were calculated using 2^−ΔΔCT^ Method (Pfaffl, 2001).

### Statistics

All data were analyzed utilizing two-way ANOVA and unpaired t-test in GraphPad Prism (GraphPad Software Inc., USA) to obtain a statistical analysis of the differences. The significance was considered as significant at *P* < 0.05 (*) and highly significant at *P* < 0.01 (**), *P* < 0.001 (***), and *P* < 0.0001 (****).

### Ethics Statement

All experiments were approved by the Institutional Animal Care and Use Committee of Sun Yat-sen University, concerning the handling of chicken embryos as well as animal experiments. And all experiments were performed in accordance with the relevant guidelines and regulations.

## RESULTS

### Efficiency of Monovalent and Bivalent Vaccination Against QX-like IBV

To evaluate efficiency of different vaccination strategies (monovalent and bivalent), several commercially available vaccines were applied in the current study. Day-old SPF chickens were inoculated with three monovalent vaccines (4/91, H120, LDT3) and 2 multi-monovalent vaccines (4/91 + Ma5, Ma5 + LDT3), respectively (Table 1). At 21 d of age, the vaccinated birds were challenged using a field-isolated prevalent QX-like (GI-19) IBV for evaluation of the protection rates of different vaccination strategies.

**Figure 1:**
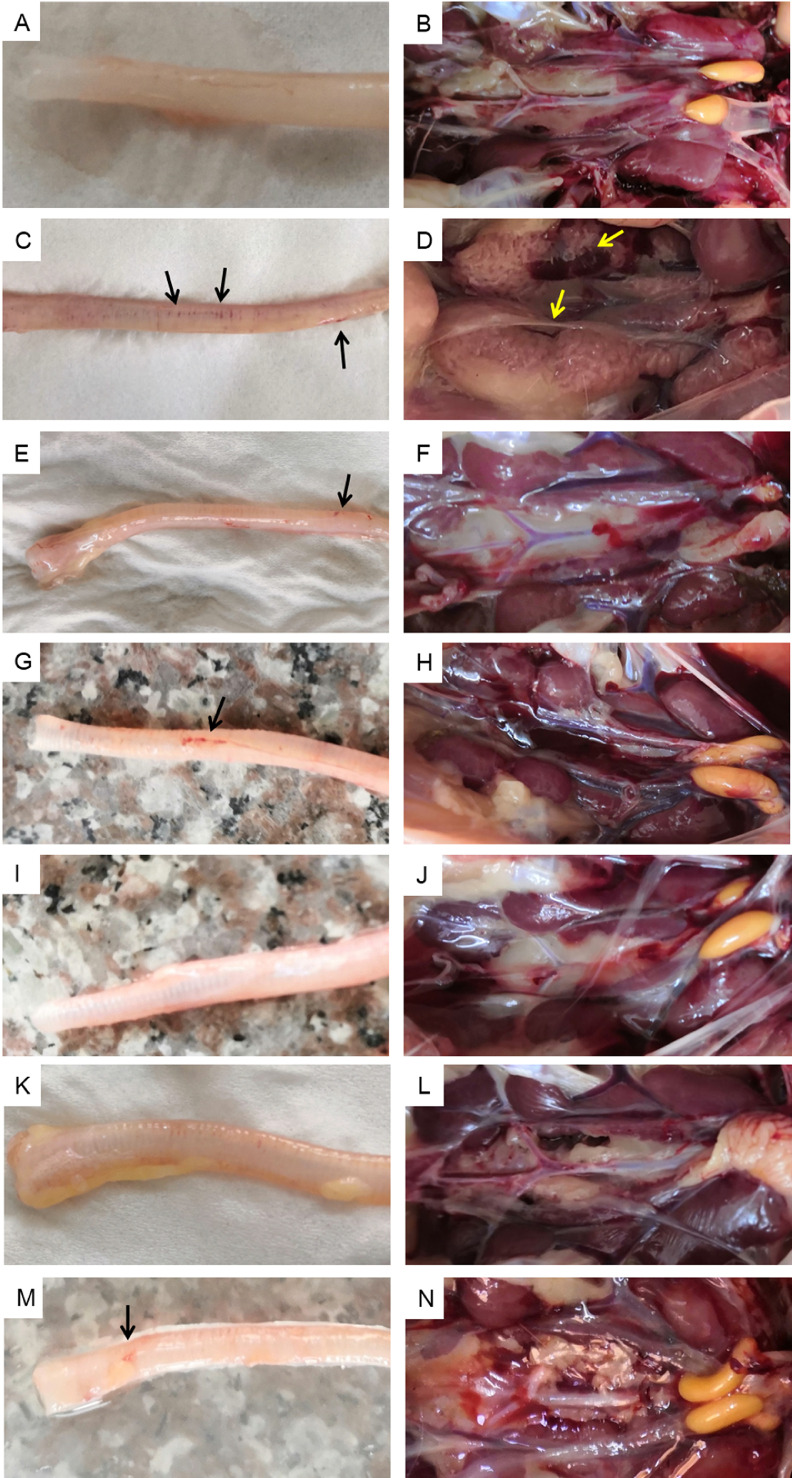
Gross lesions on kidneys and tracheas of the infected chickens after necroscopy. (A, B) Trachea and kidney of chickens in the negative control group at 7 dpc. (C, D) Trachea and kidney of infected chickens in the positive control group at 7 dpc. (E, F) Trachea and kidney of infected chickens in the 4/91 vaccine group at 7 dpc. (G, H) Trachea and kidney of infected chickens in the H120 vaccine group at 7 dpc. (I, J) Trachea and kidney of infected chickens in the LDT3 vaccine group at 7 dpc. (K, L) Trachea and kidney of infected chickens in the 4/91 + Ma5 vaccines group at 7 dpc. (M, N) Trachea and kidney of infected chickens in the Ma5 + LDT3 vaccines group at 7 dpc. Black arrow indicates lesions on trachea. Yellow arrow indicates distention with uric acid deposits in the kidney.

During the experiment, chickens in the negative control group remained healthy. The tracheas and kidneys did not show any signs of diseases (Figure 1A, B). In the positive control group, birds challenged with QX-like IBV presented mild clinical signs from 3 dpc, and 1 bird died on 7 dpc (Table 1). After necroscopy, chickens in the positive control group presented gross lesions in kidney as well as severe tracheitis. The trachea showed bleeding points and kidneys were swell with typical pale and marbled signs (Figure 1C, D). In the vaccination groups, all bird did not present any typical clinical symptoms and survived until the end of the experiment. After necroscopy, 2 birds in the 4/91 group, 4 birds in the H120 group, 4 birds in the LDT3 group presented mild lesion in kidney as well as mild tracheitis. For the 4/91 group, mild bleeding was found in the tracheas (Figure 1E), while typical nephritis characterized by pale and marbled kidneys with urate deposits in the ureters and cloaca was not found in the infected birds (Figure 1F). In the combined-vaccination groups, 3 birds in the 4/91 + Ma5 and 2 birds in LDT3 + Ma5 presented mild gross in kidney and trachea (Table 1). Mild tracheal bleeding points were found in the bivalent vaccination groups (Figure 1K, M).

Taken together, the 4/91 vaccination group presented a protection rate of 80% against QX-like (GI-19) IBV infection, while other 2 groups (H120 and LDT3) both presented a protection rate of 60% (6/10). The protective rate of 4/91+Ma5 group was 70% (7/10), and that of Ma5+LDT3 bivalent vaccine group was 80% (8/10). These data suggest monovalent 4/91 and bivalent Ma5 + LDT3 could provide efficient protection against QX-like (GI-19) IBV infection.

### IBV Antibody Levels in Serum After Monovalent or Bivalent Vaccination

To determine the antibody levels in chicken serum after monovalent and bivalent vaccination, sera of the vaccinated chickens were collected after 21 d of immunization, and serum antibody levels were detected by ELISA. Monovalent live attenuated vaccines usually can boost antibody levels as well as the multi-monovalent vaccines (Jackwood et al., 2020).

**Figure 2:**
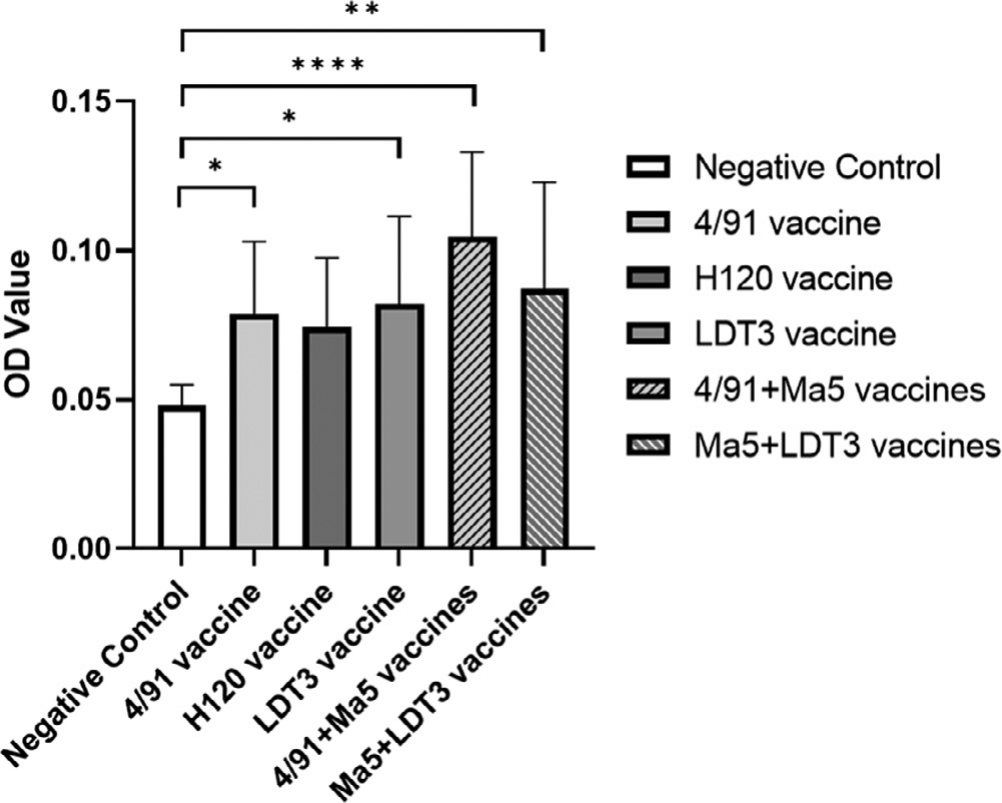
Antibody level in serum at 21 days after vaccination. All data are presented as mean ± standard deviation (SD) (n = 10); * indicates significant at *P* ≤ 0.05. ** indicates significant at *P* ≤ 0.01. **** indicates significant at *P* ≤ 0.0001.

After vaccination, the antibody level in the serum were statistically significantly upregulated in both monovalent and multi-monovalent vaccination groups, except the H120 group (Figure 2). In the H120 group, the antibody level was enhanced, while no statistical significance was observed (Figure 2). Comparing monovalent and bivalent vaccination groups, 4/91 + Ma5 group presents a higher antibody level, without statistical significance (Figure 2).

### Viral Loads and Histopathologic Changes in Kidneys and Tracheas of Monovalent and Bivalent Vaccination

**Figure 3:**
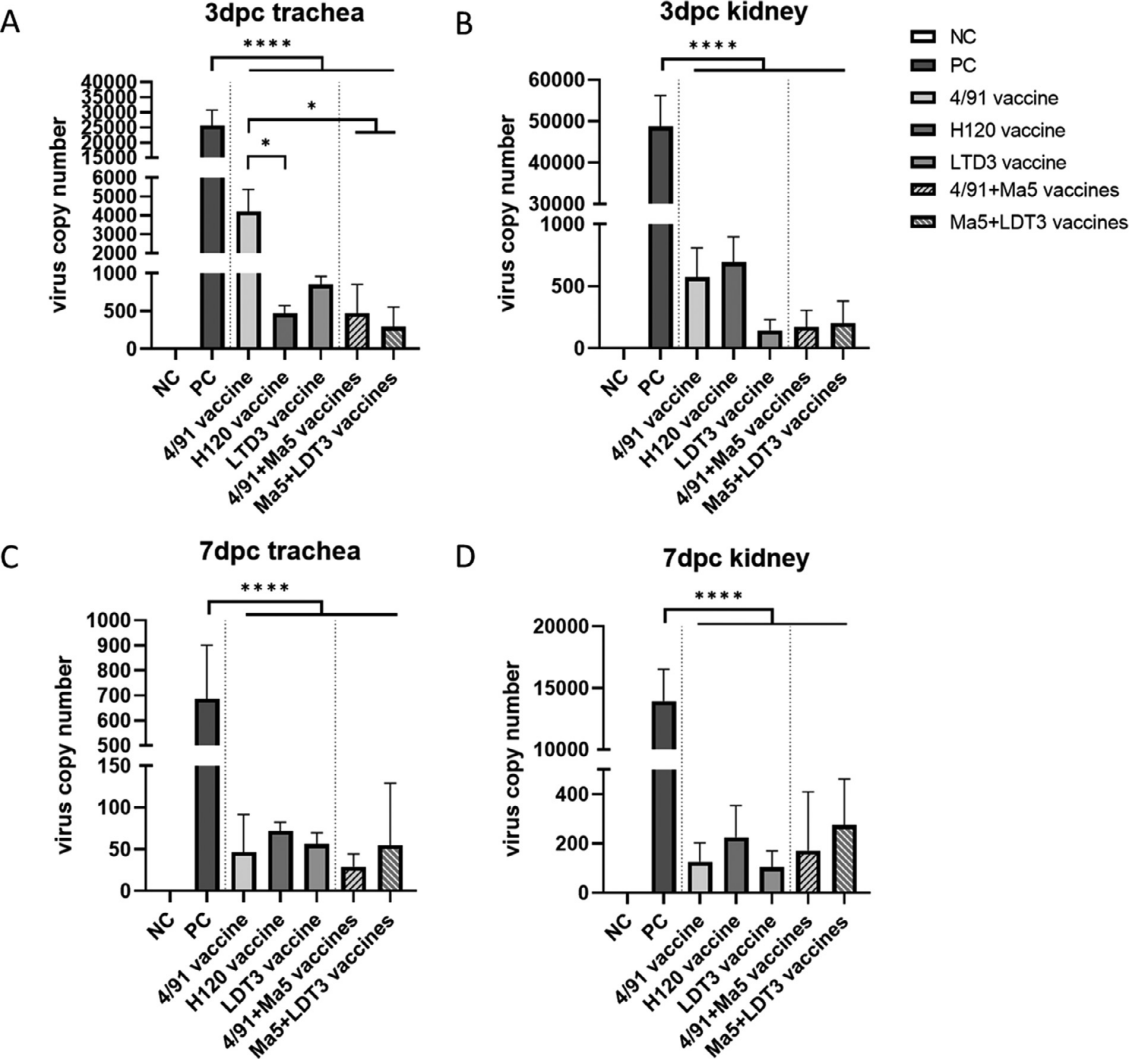
Viral loads in different tissues after infection. (A) Viral loads in tracheas of different groups at 3 dpc. (B) Viral loads in kidneys of different groups at 3 dpc. (C) Viral loads in tracheas of different groups at 7 dpc. (D) Viral loads in kidneys of different groups at 7 dpc. All data are presented as mean ± standard deviation (SD) (n = 5); * indicates significant at *P* ≤ 0.05. **** indicates significant at *P* ≤ 0.0001.

To analyze the effect of monovalent or bivalent vaccination on virus replication in different organs, tracheas and kidneys were collected at different time points (3 dpc and 7 dpc). At 3 dpc, all vaccinated groups presented significantly reduced IBV viral loads in tracheas and kidneys (*P* < 0.0001) (Figure 3A and B). The bivalent vaccination groups (4/91 + Ma5 and Ma5 + LDT3) presented lower viral loads at 3 dpc, compared to the monovalent 4/91 vaccination groups *(P* = 0.0236, *P* = 0.0481, respectively) (Figure 3A). At 7 dpc, in all vaccinated groups, viral loads in the tracheas and kidneys were significantly reduced, compared to the positive control group (*P* < 0.0001) (Figure 3C and D). The viral loads in tracheas and kidneys of the 4/91 group were lower compared to other vaccination groups, while no significant difference was observed.

**Figure 4:**
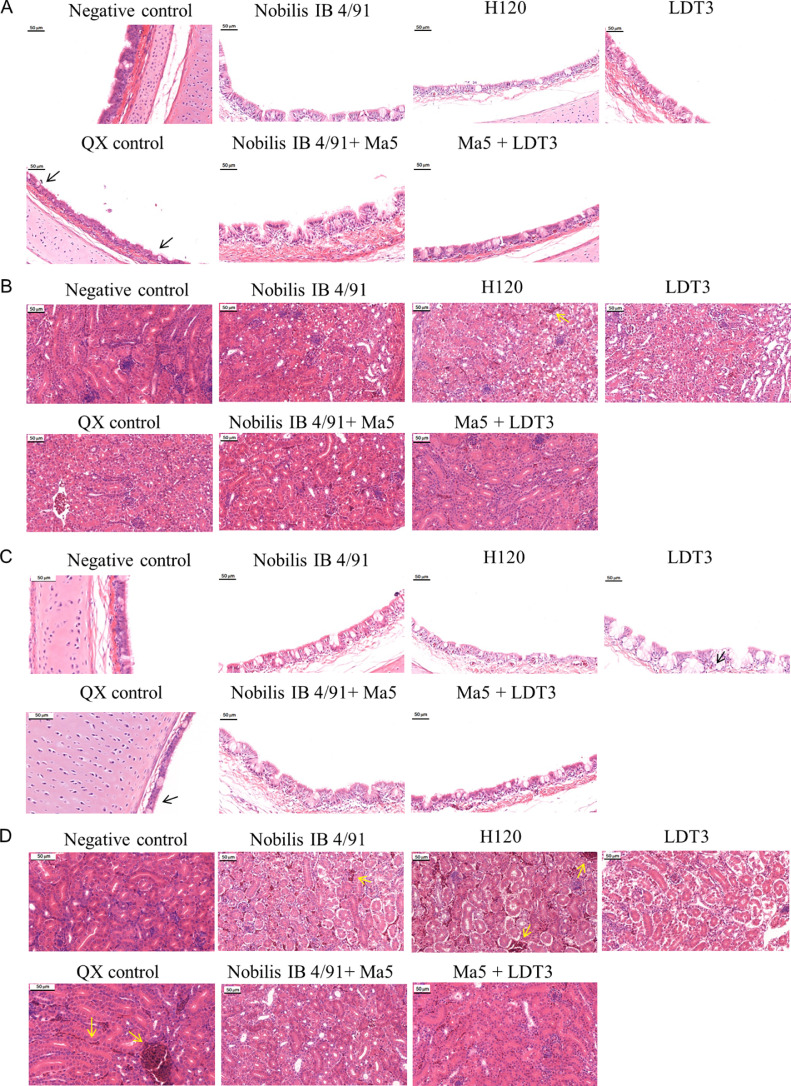
Histopathological changes in different tissues after infection. (A) Histopathological changes in tracheas of different groups at 3 dpc. (B) Histopathological changes in kidneys of different groups at 3 dpc. (C) Histopathological changes in tracheas of different groups at 7 dpc. (D) Histopathological changes in kidneys of different groups at 7 dpc. Black arrow indicates lesions on trachea cilia. Yellow arrow indicates abnormal bleeding in the kidney. Scale bar = 50 μm.

Furthermore, histopathological changes in different organs (trachea and kidney) were examined at different time points (3 dpc and 7 dpc). On 3 dpc, trachea cilia damage and desquamating were observed in the positive control group, as well as bleeding and infiltration with inflammatory cells in the kidney (Figure 4A, B). In the vaccination groups, tracheal ciliary damage was reduced, mild hemorrhage and less inflammatory cell infiltration was found in kidneys. On 7 dpc, the symptoms of tracheas and kidneys were worsened in the positive control group, while the symptoms remained mild in the vaccination groups (Figure 4C, D). Taken together, these data further suggest that monovalent 4/91 and multi-monovalent Ma5 + LDT3 could provide efficient protection against QX-like (GI-19) IBV infection.

## DISCUSSION

As a single stranded RNA virus, limited proofreading capacity of viral RNA-dependent RNA polymerase results in a high mutation rate in the IBV viral genome. The average mutation rate of coronaviruses is approximately 1.2  ×  10^−3^ substitutions/site/y (Hanada et al., 2004), while the evolutionary rate of the IBV S1 gene is 2.93  ×  10^−5^ substitutions/site/y (Zhao et al., 2016). Moreover, high recombination rate in the viral genome also contribute to broad genetic diversity of the virus (Jackwood et al., 2020).

Live attenuated vaccine vaccination is one of the main methods for IBV prevention. Traditional M41 vaccine and H120 vaccine have been widely used in the disease control. However, the high variety of the IBV genomes reduce the vaccine protection rate of the newly emerged IBV virus strains. Furthermore, vaccination with single vaccine usually can only provide limited protection to IBVs of different serotypes/genotypes, resulting in newly emerging IBV variant strains and huge difficulties in elimination and control of the disease (Bande et al., 2017), take the epidemic QX-like (GI-19) IBV strains in China for instance (Han et al., 2011; Feng et al., 2018).

Several alternative options have been applied to provide broad protection against variant IBV strains, including recombinant vaccine (Li et al., 2016), nanoparticle-based vaccine (Li et al., 2018), epitope-based vaccines (Qin et al., 2021), etc., but they have yet to be administrated with mass hatchery application. In addition, considering multi-monovalent live attenuated vaccines, though several research showed efficacy in control different IBV strains in a laboratory scale (Shao et al., 2020; Abdel-Sabour et al., 2021), it still raises many issues including the frequency of combination among field and vaccine strains (Bali et al., 2021). Therefore, proper selection of the vaccine and vaccination strategy is highly important to control the disease in the field.

To select proper vaccine candidates, in this research, we applied monovalent vaccines with genotypes of 4/91 (GI-13), Mass (GI-1), and LDT3 (GI-28) to explore the protection rate of young chickens against QX-like (GI-19) IBVs. These genotypes are prevalent in recent years in China (Han et al., 2011; Feng et al., 2018), while the QX-like (GI-19) IBVs are also prevalent in other countries (Khataby et al., 2016; Ismail et al., 2020). Bivalent vaccines (4/91+Ma5; Ma5+LDT3) were applied for evaluation as well, while bivalent vaccines with genotypes of LDT3 (GI-28) and 4/91 (GI-13) would fail to provide proper protection with emerging novel recombinant IBV strain (Gong et al., 2022). In addition, novel recombinant IBV strain with high virulence was isolated from H120 (GI-1) and 4/91 (GI-13) vaccinated flocks (Zhou et al., 2017), thus we applied Ma5 (GI-1) instead of H120 in the bivalent vaccination experiment.

After vaccination, our results showed that both monovalent and bivalent vaccination could induce antibody production, in which the bivalent 4/91 + Ma5 vaccination induced higher antibody level though statistically not significant (Figure 2). In general, bivalent vaccination groups had better performance compared to the monovalent vaccination groups, which is consistent with other research suggesting bivalent vaccination with strains of different serotypes/genotypes could provide across–protection against IBV infection (Cook et al., 1999; de Wit et al., 2011). One exception is the Nobilis IB 4/91 mono-vaccination group with a protection rate of 80%, which is higher than the bivalent vaccination group of 4/91 + Ma5 with the protection rate of 70% (Table 1). Since ten chickens/group were used in the test to evaluate the protection rate, animal individual differences might contribute to the decreased protection rate in the bivalent vaccination group, and future work with larger size of animals is required to further confirm this result. However, at an early phase of infection (3 dpc), viral loads in the tracheas of the bivalent vaccination groups were significantly lower than that of the 4/91 vaccination group, suggesting a better protection of the bivalent vaccination than the monovalent vaccination (Figure 3A). On 7 dpc, restriction of the virus proliferation was shown in both tracheas and kidneys of the vaccinated groups, comparing to the positive control group (Figure 3C, D).

Taken together, our results suggest that Nobilis IB 4/91 vaccine and bivalent vaccine had better immune protection effect, while monovalent H120 and LDT3 vaccine had poor protection rate against the QX-like (GI-19) IBVs. In addition, single dose vaccination of 4/91 revealed a total of 80% protection, suggesting single dose of Nobilis IB 4/91 might provide sufficient protection against the QX-like (GI-19) IBVs in young chickens. In summary, these results revealed that usage of monovalent 4/91 or bivalent Ma5 + LDT3 can ameliorate the pathological alterations in the trachea and kidney of the challenged young chickens. These findings indicate that 4/91 alone or bivalent IBV vaccine are suitable to serve as vaccine candidate to provide efficient protection against the QX-like (GI-19) IBV strains in the field.
